# Association of endothelial nitric oxide synthase (*NOS3*) gene polymorphisms with primary open-angle glaucoma in a Saudi cohort

**DOI:** 10.1371/journal.pone.0227417

**Published:** 2020-01-08

**Authors:** Altaf A. Kondkar, Taif A. Azad, Tahira Sultan, Essam A. Osman, Faisal A. Almobarak, Saleh A. Al-Obeidan

**Affiliations:** 1 Department of Ophthalmology, College of Medicine, King Saud University, Riyadh, Saudi Arabia; 2 Glaucoma Research Chair in Ophthalmology, College of Medicine, King Saud University, Riyadh, Saudi Arabia; Oregon Health and Science University, UNITED STATES

## Abstract

**Aim:**

To investigate the association of endothelial nitric oxide synthase (*NOS3*) gene polymorphisms in patients with primary open-angle glaucoma (POAG) of Saudi origin.

**Methods:**

This case-control study included 173 patients with POAG (94 men and 79 women) and 171 controls (98 men and 73 women). Genotyping of rs2070744 (T-786C) and rs1799983 (G894T) variants of the *NOS3* gene was performed using TaqMan^®^ assay.

**Results:**

Rs1799983 genotypes showed a significant association with POAG but did not survive Bonferroni correction (p_correction_ = 0.01). The minor ‘T’ allele was significantly associated with the risk of POAG among men (p = 0.025, odds ratio (OR) = 1.77, 95% confidence interval (CI) = 1.07–2.94). Likewise, the genotypes were significantly associated with POAG among men in dominant (p = 0.030, OR = 1.92, 95% CI = 1.06–3.48) and log-additive models (p = 0.022, OR = 1.82, 95% CI = 1.08–3.07), and after adjustment for age and smoking. Genotype and allele frequencies of rs2070744 were not significantly different between POAG cases and controls, and after sex stratification. CG haplotype was significantly protective (p = 0.011, OR = 0.52, 95% CI = 0.32–0.87) and CT haplotype conferred significantly increased risk of POAG (p = 0.016, OR = 2.60, 95% CI = 1.16–5.82) among men. Rs1799983 showed trend (p = 0.054) towards risk of POAG independent of age, gender, smoking, and rs2070744 polymorphism in logistic regression analysis. Both the polymorphisms showed no association with POAG phenotypes such as intraocular pressure and cup/disc ratio.

**Conclusion:**

Our results suggest that the polymorphism rs1799983 and the haplotypes of rs20707440 and rs1799983 in the *NOS3* gene may significantly modulate the risk of POAG in Saudi’s, particularly among men. Further larger studies are needed to confirm these findings.

## Introduction

Primary open-angle glaucoma (POAG) is a complex optic neuropathy and a significant cause of permanent blindness worldwide, including in Saudi Arabia [1, 2]. POAG pathogenesis involves damage to the optic nerve head and progressive loss of retinal ganglion cells (RGCs) that may subsequently lead to loss of vision if untreated [1]. Age, ethnicity, elevated intraocular pressure (IOP), myopia, corneal thickness, and family history are well-established risk factors of POAG [3]. Besides, there is growing evidence of compromised microvasculature [4–6] and genetic components [3, 7, 8] that may pose a potential risk for the development of POAG.

Nitric oxide (NO) is an active biological messenger that plays a key role in the regulation of vascular homeostasis and is involved in diverse physiological processes [9]. Endothelial nitric oxide synthase (eNOS), an enzyme that catalyzes the conversion of L-arginine to L-citrulline to produce NO, is an important regulator of IOP [10, 11]. Early studies in the human eye have identified extensive system of NO-producing cells in the conventional outflow pathway suggesting trabecular meshwork (TM) as an important site of NO synthesis [12]. However, newer evidence indicate that NO synthesis is predominantly localized to the Schelmm’s canal cells, where as, the TM is probably a major site of action [13], wherein the diffused NO activates the downstream signaling via soluble guanylate cyclase and cyclic guanosine monophosphate thereby contributing to vasodilatation, increase local blood flow, and decrease vascular outflow resistance in ocular circulation [12, 14–16]. Besides, NO also plays a protective role in oxidative stress-induced tissue injury or cell death [17]. eNOS may become dysfunctional as a result of constant exposure to oxidative stress leading to NO-insufficiency trigerring a cascade of pathological processes [18]. Thus, variations in eNOS activity influenced by genetic variations and/or environmental factors may play a significant role in POAG pathogenesis. Many studies have reported an association between isoform eNOS-3 (*NOS3*) gene polymorphisms and risk of POAG with inconsistent findings [19–21]. Among the important single nulecotide polymorphisms (SNPs) reported in the *NOS3* (OMIM 163729) locus are rs2070744, a T-to-C promoter variant (T-786C) and rs1799983, a G-to-T variant (G894T) at codon 298 in exon 7 (Glu298Asp). Rs2070744 (T-786C) has been shown to reduce mRNA expression [22] and rs1799983 (Glu298Asp) may alter eNOS function [23]. Besides, a recent meta-analysis also showed that polymorphisms rs1799983 and rs2070744 in *NOS3* play a significant role in modulating the risk of POAG [24]. We have recently reported negative association of polymorphisms in *TMTC2* (rs7961953) [25], *PLXDC2* (rs7081455) [26], *ATOH7* (rs7916697) [27] and at locus 1q43 [28]. The aim of the present study is to investigate the effects of *NOS3* variants on the risk of POAG and determine the association between *NOS3* polymorphisms (and haplotypes) and POAG patients of Saudi origin. The study focused on the promoter polymorphism rs2070744 and the missense polymorphism rs1799983.

## Materials and methods

### Study design and participants

In a case-control genetic association study, participants of Saudi origin with a clinically confirmed diagnosis of POAG (n = 173) and healthy controls (n = 171) were recruited at King Abdulaziz University Hospital, King Saud University, Riyadh, Saudi Arabia. The inclusion-exclusion criteria of the study population have been described previously [27]. Information concerning the history of systemic diseases, family history, and smoking status were obtained from medical records or personal interviews. All the participants signed an informed consent. The study was approved by the institutional review board and research ethics committee of the College of Medicine at the King Saud University.

### Genotyping of rs2070744 and rs1799983

Genomic DNA samples from the study population were genotyped using the TaqMan^®^ SNP Genotyping Assay (Applied Biosystems Inc., Foster City, CA, USA) on ABI 7500 Real-Time PCR System (Applied Biosystems) as described previously [27]. Assay IDs:

C__15903863_10 (Catalog number: 4351379) and C___3219460_20 (Catalog number: 4351379) were used to genotype rs2070744 and rs1799983, respectively.

### Statistical analysis

Pearson’s Chi^2^ test was used to test deviation from Hardy-Weinberg Equilibrium (HWE) and associations between allele/genotype profiles. The continuous variables were tested by Independent samples *t*-test, and Kruskal-Wallis were used to test difference across genotypes. Binary logistic regression was used to test the effects of age, gender, smoking habit, and genotypes on POAG outcome. SPSS version 22 (IBM Inc. Chicago, Illinois, USA) was used to perform statistical tests. SNPStats online software (https://www.snpstats.net/start.htm) was used for SNP analyses and their interactions with related factors. SHEsis online software (http://analysis.bio-x.cn/myAnalysis.php) was used to assess linkage disequilibrium (LD) and analyze haplotypes. Power analysis was performed using an open source online tool for unmatched case-control studies (http://sampsize.sourceforge.net/iface/s3.html#ccp). A two-tailed p<0.05 was considered statistically significant. Bonferroni’s correction was applied for multiple testing and set at p<0.01 where applicable.

## Results

**Table 1** summarizes the general characteristics of POAG cases and controls included in the study. The study groups did not differ in terms of age, gender distribution, systemic disease status, and smoking habits. However, the frequency of family history of glaucoma was significantly higher in cases.

**Table 1:** Characteristics of study participants.

Characteristics	POAG (n = 173) n (%)	Controls (n = 171) n (%)	Odds ratio	95% confidence interval	p-value
Age in years (SD)	60.9 (10.9)	58.9 (11.5)	-	-0.42–4.32	0.108
Male/Female, n	94/79	98/73	0.88	0.58–1.35	0.579
Diabetes mellitus	66 (38.1)	65 (38.0)	1.00	0.65–1.55	0.979
Hypertension	65 (37.5)	56 (32.7)	1.23	0.79–1.92	0.349
Coronary artery disease	5 (2.9)	4 (2.4)	1.24	0.32–4.70	0.749
Smoking	19 (10.9)	15 (8.7)	1.28	0.63–2.61	0.492
Family history of glaucoma	17 (9.8)	7 (4.1)	2.55	1.03–6.32	0.037

**Table 2** summarizes the minor allele frequency (MAF) distribution of *NOS3* polymorphisms in cases and controls. Overall, no significant differences were observed for allele frequencies of rs2070744 and rs1799983 polymorphisms between cases and controls. However, variant rs1799983 (G984T, Glu298Asp) was significantly associated with increased risk of POAG only among men (OR = 1.77, 95% CI = 1.07–2.94, p = 0.025). No such gender-specific association was observed for rs2070744 (T-786C).

**Table 2:** Minor allele frequency of *NOS3* polymorphisms in POAG cases and controls.

SNP Group	Cases MAF	Controls MAF	Odds ratio (95% confidence interval)	p-value
rs2070744				
Total	0.32	0.32	0.98 (0.71–1.35)	0.916
Men	0.27	0.31	0.82 (0.53–1.28)	0.389
Women	0.37	0.34	1.18 (0.73–1.89)	0.491
rs1799983				
Total	0.23	0.18	1.38 (0.95–2.01)	0.086
Men	0.25	0.16	1.77 (1.07–2.94)	**0.025**
Women	0.21	0.20	1.02 (0.58–1.77)	0.920

Abbreviation: MAF, minor allele frequency.

Note: Significant p-value in bold.

POAG is a complex disease with no clear genetic mode of inheritance. Hence, co-dominant, dominant, recessive, over-dominant, and log-additive genetic models were used to test for association between SNPs in the *NOS3* gene and the risk of POAG using SNPStats software. The results of these genetic models are shown in **Table 3**. We found that rs1799983 of the *NOS3* gene was associated with POAG risk (Table 3) in dominant and over-dominant models with lowest Akaike’s information criterion (AIC) and Bayesian information criterion (BIC) values indicating the best-fit model. The p-value remained significant after adjustment for age and sex. However, the significance was lost after Bonferroni correction (*p*_correction_ = 0.05/5 = 0.01). On the other hand, SNP rs2070744 did not show any significance in the tested genetic models (Table 3).

**Table 3:** Association of *NOS3* polymorphisms with the risk of POAG compared to control under different genetic models.

SNP number	Model	Genotype	CONTROL n (%)	POAG n (%)	Odds ratio (95% confidence interval)	p-value	AIC	BIC	p-value^§^
rs2070744	Co-dominant	T/T	75 (43.9)	81 (46.8)	1.00	0.570	481.7	493.3	0.480
		C/T	82 (48.0)	74 (42.8)	0.84 (0.54–1.30)				
		C/C	14 (8.2)	18 (10.4)	1.19 (0.55–2.56)				
	Dominant	T/T	75 (43.9)	81 (46.8)	1.00	0.580	480.6	488.3	0.540
		C/T-C/C	96 (56.1)	92 (53.2)	0.89 (0.58–1.36)				
	Recessive	T/T-C/T	157 (91.8)	155 (89.6)	1.00	0.480	480.4	488.1	0.410
		C/C	14 (8.2)	18 (10.4)	1.30 (0.63–2.71)				
	Over-dominant	T/T-C/C	89 (52.0)	99 (57.2)	1.00	0.330	479.9	487.6	0.280
		C/T	82 (48.0)	74 (42.8)	0.81 (0.53–1.24)				
	Log-additive	---	---	---	0.98 (0.71–1.36)	0.910	480.9	488.5	0.920
rs1799983	Co-dominant	G/G	117 (68.4)	100 (57.8)	1.00	0.104	478.3	489.9	0.095
		G/T	47 (27.5)	66 (38.1)	1.64 (1.04–2.60)				
		T/T	7 (4.1)	7 (4.0)	1.17 (0.40–3.45)				
	Dominant	G/G	117 (68.4)	100 (57.8)	1.00	0.041*	476.7	484.4	**0.035**^†^
		G/T-T/T	54 (31.6)	73 (42.2)	1.58 (1.02–2.46)				
	Recessive	G/G-G/T	164 (95.9)	166 (96)	1.00	0.98	480.9	488.6	0.95
		T/T	7 (4.1)	7 (4.0)	0.99 (0.34–2.88)				
	Over-dominant	G/G-T/T	124 (72.5)	107 (61.9)	1.00	0.035*	476.4	484.1	**0.033**^‡^
		G/T	47 (27.5)	66 (38.1)	1.63 (1.03–2.56)				
	Log-additive	---	---	---	1.39 (0.95–2.03)	0.084	477.9	485.6	0.070

^§^Adjusted for age, sex and smoking

^†^OR (95% CI) = 1.61 (1.03–2.52)

^‡^OR (95% CI) = 1.64 (1.04–2.60)

*Best-fit model *p*-value

Abbreviations: AIC, Akaike’s information criterion; BIC, Bayesian information criterion.

Note: Significant p-value in bold.

A similar gender-stratified genotype analysis for rs2070744 and rs1799983 are summarized in **Table 4** and **Table 5**. The variant genotype of rs1799983 significantly increased the risk of POAG by almost ~2-folds in dominant and log-additive models among men (Table 4). The association remained significant after adjustment for age and sex, but not for Bonferroni correction. No such association was observed among the women group, thus indicating that the polymorphism rs1799983 were significantly associated with the risk of POAG only among men (Table 4). Besides, polymorphism rs2070744 did not show any gender-specific association (Tables 4 and 5).

**Table 4:** Association testing of *NOS3* polymorphisms under different genetic models among men.

SNP number	Model	Genotype	CONTROL n (%)	POAG n (%)	Odds ratio (95% confidence interval)	p-value	AIC	BIC	p-value^§^
rs2070744	Co-dominant	T/T	43 (43.9)	49 (52.1)	1.00	0.480	270.6	280.4	0.430
		C/T	49 (50.0)	39 (41.5)	0.70 (0.39–1.26)				
		C/C	6 (6.1)	6 (6.4)	0.88 (0.26–2.92)				
	Dominant	T/T	43 (43.9)	49 (52.1)	1.00	0.250	268.8	275.3	0.220
		C/T-C/C	55 (56.1)	45 (47.9)	0.72 (0.41–1.27)				
	Recessive	T/T-C/T	92 (93.9)	88 (93.6)	1.00	0.940	270.1	276.6	0.890
		C/C	6 (6.1)	6 (6.4)	1.05 (0.32–3.36)				
	Over-dominant	T/T-C/C	49 (50.0)	55 (58.5)	1.00	0.240	268.7	275.2	0.200
		C/T	49 (50.0)	39 (41.5)	0.71 (0.40–1.25)				
	Log-additive	---	---	---	0.80 (0.50–1.29)	0.360	269.3	275.8	0.340
rs1799983	Co-dominant	G/G	69 (70.4)	52 (55.3)	1.00	0.074	266.9	276.6	0.051
		G/T	27 (27.6)	37 (39.4)	1.82 (0.99–3.36)				
		T/T	2 (2.0)	5 (5.3)	3.32 (0.62–17.78)				
	Dominant	G/G	69 (70.4)	52 (55.3)	1.00	0.030*	265.4	271.9	**0.019**^†^
		G/T-T/T	29 (29.6)	42 (44.7)	1.92 (1.06–3.48)				
	Recessive	G/G-G/T	96 (98.0)	89 (94.7)	1.00	0.220	268.6	275.1	0.210
		T/T	2 (2.0)	5 (5.3)	2.70 (0.51–14.25)				
	Over-dominant	G/G-T/T	71 (72.5)	57 (60.6)	1.00	0.082	267.1	273.6	0.057
		G/T	27 (27.6)	37 (39.4)	1.71 (0.93–3.13)				
	Log-additive	---	---	---	1.82 (1.08–3.07)	0.022*	264.9	271.4	**0.015**^‡^

^§^Adjusted for age and smoking

^†^odds ratio (OR) (95% confidence interval [CI]) = 2.03 (1.12–3.73)

^‡^OR (95% CI) = 1.90 (1.12–3.25)

*Best-fit model *p*-value

Abbreviations: AIC, Akaike’s information criterion; BIC, Bayesian information criterion.

Note: Significant p-value in bold.

**Table 5:** Association testing of *NOS3* polymorphisms under different genetic models among women.

SNP number	Model	Genotype	CONTROL n (%)	POAG n (%)	Odds ratio (95% confidence interval)	p-value	AIC	BIC	p-value^§^
rs2070744	Co-dominant	T/T	32 (43.8)	32 (40.5)	1.00	0.730	215.9	224.9	0.670
		C/T	33 (45.2)	35 (44.3)	1.06 (0.54–2.10)				
		C/C	8 (11.0)	12 (15.2)	1.50 (0.54–4.16)				
	Dominant	T/T	32 (43.8)	32 (40.5)	1.00	0.680	214.3	220.4	0.690
		C/T-C/C	41 (56.2)	47 (59.5)	1.15 (0.60–2.18)				
	Recessive	T/T-C/T	65 (89.0)	67 (84.8)	1.00	0.440	213.9	219.9	0.370
		C/C	8 (11.0)	12 (15.2)	1.46 (0.56–3.79)				
	Over-dominant	T/T-C/C	40 (54.8)	44 (55.7)	1.00	0.910	214.5	220.5	0.850
		C/T	33 (45.2)	35 (44.3)	0.96 (0.51–1.83)				
	Log-additive	---	---	---	1.18 (0.74–1.87)	0.500	214	220.1	0.470
rs1799983	Co-dominant	G/G	48 (65.8)	48 (60.8)	1.00	0.250	213.7	222.8	0.280
		G/T	20 (27.4)	29 (36.7)	1.45 (0.72–2.91)				
		T/T	5 (6.8)	2 (2.5)	0.40 (0.07–2.16)				
	Dominant	G/G	48 (65.8)	48 (60.8)	1.00	0.520	214.1	220.1	0.52
		G/T-T/T	25 (34.2)	31 (39.2)	1.24 (0.64–2.40)				
	Recessive	G/G-G/T	68 (93.2)	77 (97.5)	1.00	0.200	212.8	218.9	0.220
		T/T	5 (6.8)	2 (2.5)	0.35 (0.07–1.88)				
	Over-dominant	G/G-T/T	53 (72.6)	50 (63.3)	1.00	0.220	213	219	0.230
		G/T	20 (27.4)	29 (36.7)	1.54 (0.77–3.06)				
	Log-additive	---	---	---	1.02 (0.59–1.77)	0.940	214.5	220.5	0.920

^§^Adjusted for age

Abbreviations: AIC, Akaike’s information criterion; BIC, Bayesian information criterion.

The genotype frequencies of rs2070744 and rs1799983 were consistent with HWE for both cases and controls among overall, men and women groups.

The two SNPs were tested for LD and haplotype analyses using the SHEsis software platform. The standardized LD coefficient D’ value between rs2070744 and rs1799983 was 0.14 (r^2^ = 0.011), indicating these SNPs are not in high LD. **Table 6** shows the analyses based on haplotypes of polymorphisms rs2070744 and rs1799983. Overall, none of the haplotypes showed any significant effect on the risk of POAG. However, consistent with the above genotype results, the haplotype distribution showed significant distribution among men (*X*^2^ = 10.815, df = 3, p = 0.012). Haplotype CG was found to be protective against POAG (p = 0.011, OR = 0.52, 95% CI = 0.32–0.87); whereas, haplotype CT was related to a significantly higher risk of POAG among men only (p = 0.016, OR = 2.60, 95% CI = 1.16–5.82).

**Table 6:** Haplotype analysis of *NOS3* polymorphisms.

Haplotypes*	Cases, Frequency	Control, Frequency	Odds ratio (95% confidence interval)	p-value
**TOTAL**				
TG	0.55	0.57	0.90 (0.66–1.22)	0.500
CG	0.22	0.25	0.85 (0.60–1.22)	0.395
TT	0.13	0.10	1.32 (0.83–2.11)	0.236
CT	0.09	0.07	1.35 (0.79–2.31)	0.269
**Men**^†^				
TG	0.59	0.58	1.05 (0.70–1.58)	0.787
CG	0.16	0.26	0.52 (0.32–0.87)	**0.011**
TT	0.14	0.11	1.27 (0.69–2.33)	0.434
CT	0.11	0.04	2.60 (1.16–5.82)	**0.016**
**Women**				
TG	0.50	0.56	0.78 (0.50–1.23)	0.296
CG	0.29	0.23	1.33 (0.80–2.23)	0.265
TT	0.13	0.10	1.24 (0.61–2.51)	0.551
CT	0.08	0.10	0.79 (0.36–1.75)	0.572

*in the order of rs2070744-rs1799983

^†^Overall Chi-square = 10.815, df = 3, p = 0.012. Note: Significant p-value in bold.

**Fig 1:**
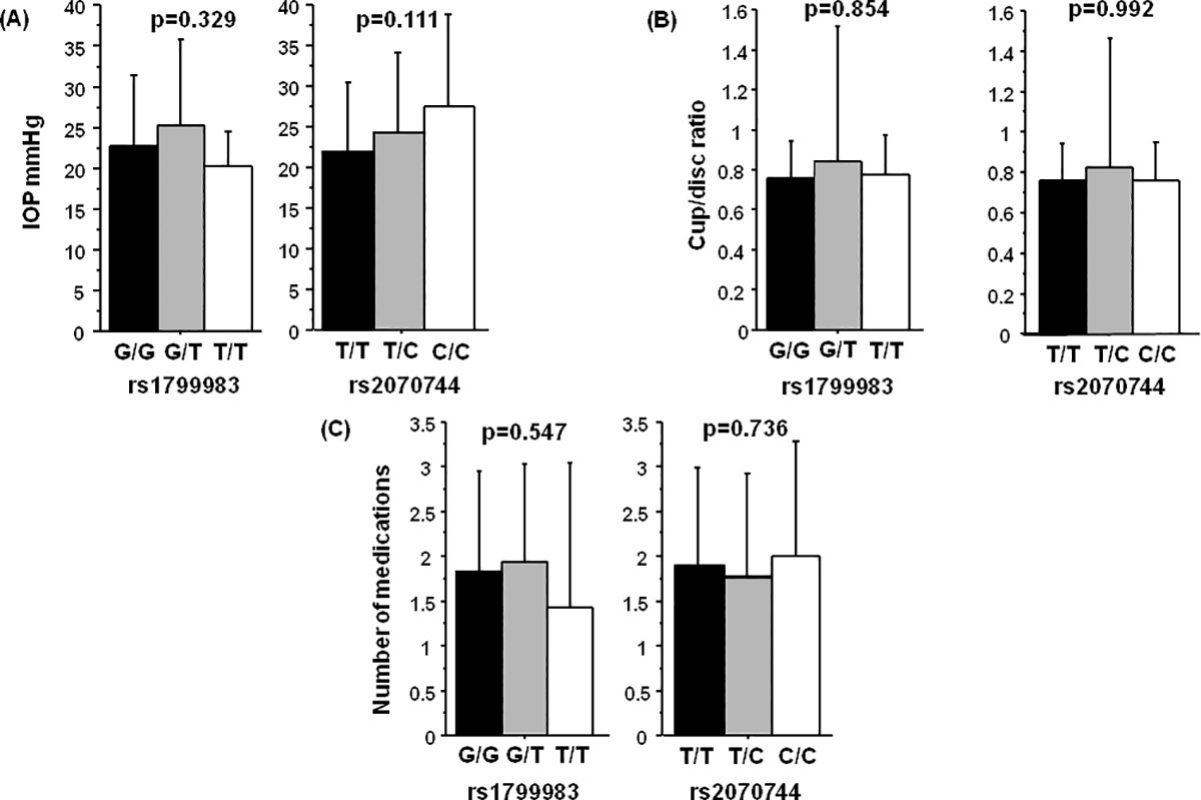
Genotype effect of rs1799983 and rs2070744 on glaucoma specific clinical indices in PAOG cases. (A) intraocular pressure (IOP), (B) cup/disc ratio and (C) number of antiglaucoma medications. Note: p-value calculated by Kruskal-Wallis test.

A binary logistic regression analysis was performed to test the effect of age, sex, smoking status, and *NOS3* polymorphisms on POAG outcome (**Table 7**). The analysis revealed that only SNP rs1799983 exhibited a trend for the independent risk factor of POAG (p = 0.054) with the heterozygous G/T genotype significantly increasing the risk of POAG (p = 0.016, OR = 1.79, 95% CI = 1.11–2.89). Also, no genotype-specific association was observed with POAG related clinical phenotypes such as IOP, cup/disc ratio, and the number of antiglaucoma medications for both the polymorphisms (**Fig 1**).

**Table 7:** Binary logistic regression analysis to determine the effect of *NOS3* polymorphisms, age, sex and smoking on POAG risk.

Variables	B	SE	Wald	Odds ratio (95% confidence interval)	p-value
Age	0.02	0.01	3.60	1.02 (0.99–1.039)	0.058
Sex	-0.11	0.23	0.23	0.89 (0.56–1.41)	0.630
Smoking	0.31	0.39	0.66	1.37 (0.64–2.93)	0.416
rs2070744	-	-	2.66	-	0.265
C/T	-0.30	0.23	1.63	0.74 (0.46–1.17)	0.202
C/C	0.25	0.40	0.40	1.28 (0.58–2.82)	0.529
rs1799983	-	-	5.82	-	**0.054**
G/T	0.58	0.24	5.82	1.79 (1.11–2.89)	**0.016**
T/T	0.21	0.56	0.14	1.23 (0.41–3.70)	0.709
Constant	-1.19	0.66	3.26	0.30	0.071

Trend and significant p-value in bold

## Discussion

Several theories have been proposed to explain the glaucomatous optic nerve degeneration [29]. Endogenous NO signaling within the conventional outflow pathway is one of the key mediators of outflow regulation and maintenance of physiological levels of IOP [10, 12]. Alterations in NO signaling or reduced NO production in part may result in impaired trabecular outflow and contribute to IOP elevation in POAG [10, 13]. Likewise, the role of eNOS/NO pathway in the maintenance of ocular vasculature and optic nerve degeneration is also highly critical [29]. Notably, functional polymorphisms in the *NOS3* gene that alter eNOS function/activity have been described in various complex diseases, including glaucoma [21]. In the present study, we report a significant association between *NOS3* polymorphism rs1799983 (and rs2070744 haplotypes) in POAG patients of Saudi origin.

*NOS3* gene is located in 7q35-q36 encoding a protein consisting of 1,203 amino acids. The frequency of *NOS3* allelic variants, rs1799983 and rs2070744, appear to vary among various population groups. The MAF of rs1799983 varies from 0.34 in European to 0.13 in Asian and 0.07 in African populations (dbSNP database). Similarly, the rs2070744 MAF is 0.43 among Europeans and 0.12–01.3 in African and Asians (dbSNP database). The MAF for both these SNPs (rs1799983 and rs2070744) observed in our study (Saudi Arabians) is lower than the European but higher than the African and Asian populations, suggesting an ethnic-specific distribution.

Yoshimura et al. described the Glu298Asp (rs1799983) missense variant to be associated with coronary spasm in the Japanese population [30]. Subsequent investigations described other polymorphisms in the 5' flanking and some introns of the *NOS3* gene. Likewise, a promoter variant in the *NOS3* gene exhibited significant link in patients with familial POAG [31]. Since then, several studies have evaluated the association of these variants, particularly rs1799983 (G894T) and rs2070744 (T-786C) in POAG with conflicting results. A comparison of these studies in different ethnic groups with our study is listed in **Table 8**. It can be noted that though negative associations are also reported [19, 32], in most of the investigations it is the promoter polymorphism (rs2070744) that was associated with POAG. Besides, these associations were of significance in women [20, 21, 33], influenced by hormone use [33], blood pressure [34], cigarette smoking [34], or migraine [35] suggestive of an epistatic interaction(s). In contrast, our study reports a positive association of rs1799983 (G894T, Glu298Asp) with POAG, particularly among men with no effect of age, gender, or smoking habits on genotype distribution.

**Table 8:** Comparison of association studies of *NOS3* investigating rs2070744 and rs1799983 polymorphisms in different population.

Population	Sample size/ Type	MAF rs2070744 (T-786C)	MAF rs1799983 (G894T)	Findings	Reference
Chinese	66 POAG 100 Control	0.08 POAG 0.08 Control	Not studied	No association	[19]
European	117 Glaucoma 24 h/o migrane 36 Control	0.38 Glaucoma 0.35 h/o migraine 0.39 Control	Not studied	Haplotype including T-786C associated with glaucoma who have a h/o migraine	[35]
Caucasian	527 POAG 1539 Control	0.40 POAG 0.38 Control	0.35 POAG 0.32 Control	Interaction of rs2070744 with females and hormone use in HTG; and rs2070744 was associated with hypertension	[33, 34]
Chinese	405 POAG 201 Control	0.12 POAG 0.12 Control	0.12 POAG 0.11 Control	No association	[32]
Brazilian	90 POAG 127 Control	0.31 POAG 0.24 Control	0.23 POAG 0.24 Control	rs2070744 genotype associated with POAG among women	[21]
Egyptian	160 HT-POAG 110 Control	0.42 POAG 0.28 Control	0.31 POAG 0.26 Control	rs2070744 associated with HTG, particularly among women	[20]
Saudi	173 POAG 171 Control	0.32 POAG 0.32 Control	0.23 POAG 0.18 Control	rs1799983 (and rs2070744 haplotype) associated with POAG among men	This study

Abbreviations: MAF, minor allele frequency; POAG, primary open-angle glaucoma; h/o, history of; HT-POAG, high-tension primary open-angle glaucoma

Similarly, the role of these polymorphisms has also been examined in normal-tension glaucoma (NTG) with both positive and negative associations. In a Polish study, rs1799983 showed marginal association with NTG; and both rs1799983 and rs2070744 were associated with low systolic blood pressure in NTG [36]. Jeoung et al. reported a significant association of rs2070744 in Korean NTG patients with optic disc hemorrhage [37]; whereas both these SNPs showed no association with NTG or high-tension glaucoma in German Caucasians [38]. Also, the promoter SNP showed an increased prevalence in patients with non-arteritic anterior ischemic optic neuropathy [39].

There can be several potential reasons for different association findings. These differences could be attributable to population-stratification or selection bias. Ours being a tertiary care center, there can be a referral bias and may not reflect the general population. The association could reflect an ethnic-specific genetic etiology in POAG. Differences in environmental exposures, different non-*NOS3* genetic backgrounds or association with other polymorphism(s) in LD with a nearby causal or functional mutation cannot be ruled out. Finally, given the relatively small number of samples and polymorphisms tested these difference could be attributable to chance or probability.

Haplotype analyses can be more informative in genetic case-control studies of complex diseases that involve multiple susceptibility markers. Haplotype analyses of rs2070744 (T-786C) and rs1799983 (G894T) in all POAG cases and controls showed no association of haplotypes with the risk of POAG. However, further gender-stratification of haplotypes identified potential risk (CT), and protective (CG) haplotypes among men but not among women. Few studies have examined haplotype effect with the risk of POAG. In contrast to our results, a study investigating rs2070744 and rs1799983 in a Brazilian cohort, haplotype CG showed marginal association with the risk of POAG with borderline risk among women but not among men [21]. Likewise, Logan et al. reported a significant association of haplotype including T-786C (rs2070744) and microsatellite markers in the promoter region of *NOS3* with glaucoma in patients with a history of migraine [35], a condition predominant among women [40]. Besides, the CT haplotype constituted by rs3793342 and rs11771443 was a risk factor of POAG in the Han Chinese population [41]. Similarly, the TT homozygotes of G894T with at least one C allele of T-786C polymorphism were considered a more significant risk factor for coronary artery disease [42]. Haplotypes C4bT and C4bG consisting of T-786C, intron 4b/4a, and G894T SNPs have been associated with substantial 7-fold risk and a non-significant protective effect (OR = 0.56 [0.16–1.90]), respectively in dilated cardiomyopathy (a condition manifested as a result of oxidative stress that leads to heart failure) [43]; and haplotype C4bT was also reported to be a risk factor for hypertension [44]. It is difficult to ascertain whether the protective or risk effects observed in our study are attributable to a real haplotype effect or reflects a strong LD with another causal variant(s) not included in this study.

Our study shows that *NOS3* polymorphism rs1799983 (894T allele) is a significant risk factor for POAG and that haplotypes CG and CT of rs2070744 and rs1799983 can significantly modulate the risk of POAG, particularly among men only. Except for rs10483727 in the *SIX1*/*SIX6* [45], this finding is in contrast to the lack of association previously reported in our similar cohort studies in Saudi POAG patients [25–28, 46–49] in the genetic loci that significantly influenced POAG or its endophenotypes in other ethnicities [3]. *NOS3* codes for NO derived from vascular endothelium. There is increasing experimental and clinical evidence to suggest a critical etiological and therapeutic role of NO in POAG [15]. Lower levels of plasma and aqueous humor NO have been demonstrated in POAG [14]. Inhibition of NO pathway has been shown to result in decreased ocular blood flow in healthy individuals due to defective NO synthesis [50]. The C allele of *NOS3* promoter polymorphism rs 2070744 (T-786C) has been shown to reduce the transcription rate of *NOS3* and have lower *NOS3* mRNA and protein levels [20, 22]. Besides, some studies have shown that the eNOS enzyme activity decreases in the presence of rs1799983 minor 894T allele [51] and that eNOS from patients with an 894T allele are susceptible to intracellular cleavage by an unknown protease, thereby providing a possible mechanism to explain impairment in eNOS function [23]. These observations are also well supported by a high degree of linkage between T-786 and G894 alleles, at least in the Caucasians [52] as opposed to a weak linkage observed in our study. Also, it has been suggested that this substitution affects protein-protein interaction [53]; affects interaction with caveolin-1, resulting in impaired localization and eNOS activity [54]; and that healthy carriers of Glu298Asp variant exhibit functional changes in the endothelium [55].

Based on these findings, our results suggest that reduced NO production and subsequent deregulation in TM outflow pathway may be a plausible mechanism(s) contributing to the development of POAG in our cohort. Low concentrations of NO may also lead to an imbalance in redox symbiotic relationship between NO and oxidative stress and compromise the ability of NO to abate oxidative stress-induced damage that plays an essential role in both physiological and pathophysiological mechanisms of POAG [17]. Our group has previously shown that the total antioxidant status is compromised in patients with POAG and more prone to oxidative stress-induced glaucomatous neurodegeneration [56]. Though Kosior-Jarecka et al. suggested a significant correlation between *NOS3* polymorphisms and IOP in patients with high-tension glaucoma [36], our study however, did not observe any similar effect between *NOS3* polymorphisms and clinical markers of POAG such as IOP and cup/disc ratio. Also, absence of any association in women is difficult to explain and may probably be due to low numbers in this group.

The functional role of rs1799983 (Glu298Asp) is, however, controversial. There are in vivo and in vitro studies that have provided contrasting evidence to suggest that Glu298Asp does not affect the biological activity or function of eNOS [57–59]. However, it is noteworthy that specific haplotypes and cigarette smoking have been shown to influence transcription efficiency and affect eNOS activity [51, 60], highlighting a significant role for gene-gene or gene-environmental interaction in eNOS regulation. Besides, the presence of a strong LD with an actual functional or causal variant elsewhere in the gene or genome cannot be ruled out.

The study has few limitations and requires cautious interpretation. The sample size examined in this study is relatively small, with even fewer numbers in subgroup analyses. Nonetheless, based on the allele frequency observed in our cohort and assuming an OR of 2.0 with one-sided test at an alpha-risk of 5%, the study exhibited powers of 0.86 and 0.93 to detect significant associations between POAG and polymorphisms rs1799983 and rs2070744, respectively. However, the study was not significantly powered to detect an OR of 1.5 or less. As in most genetic association studies of complex diseases, detecting an OR ≤1.5 would require investigating a susbtantially larger population. Such may be the case for lack of association with rs2070744 polymorphism or among women. There could be a referral or selection bias in the study and may not reflect the general Saudi population. Only two *NOS3* polymorphisms were examined. Investigation of other variant (s), e.g., intron4b/4a (repeat polymorphism) or estimation of NO concentrations would have been further informative. The study does not provide any mechanistic evidence for the role of these SNPs in POAG, and hence the presence of another causal variant, gene-gene or gene-environment interactions influencing the study outcome cannot be ruled out. Assuming a critical role of epistatic interactions affecting eNOS activity and function further emphasizes the need for validation in a well-designed sizeable population-based cohort.

In conclusion, the study demonstrates a significant association of polymorphism rs1799983 and haplotypes of rs2070744 and rs1799983 in the *NOS3* gene in the modulating the risk of POAG, particularly among men of Saudi origin. A validation or replication of association studies in different ethnicity is of genetic epidemiological importance and an important tool to examine their future utility as genetic biomarkers in diagnosis or prognosis of a disease. Our findings suggest that *NOS3* polymorphism(s) may be a significant genetic biomarker in POAG and provides an additional evidence to support the hypothesis that reduced NO (or enhanced oxidative stress) may play a vital role in the pathogenesis of POAG. However, further studies with larger sample size are needed to confirm this association.

## References

[1] Kwon YH, Fingert JH, Kuehn MH, Alward WL. Primary open-angle glaucoma. N Engl J Med. 2009;360(11):1113–24. Epub 2009/03/13. 10.1056/NEJMra0804630 360/11/1113 [pii]. 19279343 PMC3700399

[2] Al Obeidan SA, Dewedar A, Osman EA, Mousa A. The profile of glaucoma in a Tertiary Ophthalmic University Center in Riyadh, Saudi Arabia. Saudi J Ophthalmol. 2011;25(4):373–9. Epub 2011/10/01. 10.1016/j.sjopt.2011.09.001 SJOPT118 [pii]. 23960951 PMC3729326

[3] Abu-Amero K, Kondkar AA, Chalam KV. An Updated Review on the Genetics of Primary Open Angle Glaucoma. Int J Mol Sci. 2015;16(12):28886–911. Epub 2015/12/23. 10.3390/ijms161226135 ijms161226135 [pii]. 26690118 PMC4691082

[4] Topouzis F, Wilson MR, Harris A, Founti P, Yu F, Anastasopoulos E, et al Association of open-angle glaucoma with perfusion pressure status in the Thessaloniki Eye Study. Am J Ophthalmol. 2013;155(5):843–51. Epub 2013/02/12. 10.1016/j.ajo.2012.12.007 S0002-9394(12)00861-6 [pii]. . 23394905

[5] Orzalesi N, Rossetti L, Omboni S. Vascular risk factors in glaucoma: the results of a national survey. Graefes Arch Clin Exp Ophthalmol. 2007;245(6):795–802. Epub 2006/11/23. 10.1007/s00417-006-0457-5 . 17120006

[6] Su WW, Cheng ST, Ho WJ, Tsay PK, Wu SC, Chang SH. Glaucoma is associated with peripheral vascular endothelial dysfunction. Ophthalmology. 2008;115(7):1173–8 e1. Epub 2007/12/14. S0161-6420(07)01178-5 [pii] 10.1016/j.ophtha.2007.10.026 . 18076992

[7] Janssen SF, Gorgels TG, Ramdas WD, Klaver CC, van Duijn CM, Jansonius NM, et al The vast complexity of primary open angle glaucoma: disease genes, risks, molecular mechanisms and pathobiology. Prog Retin Eye Res. 2013;37:31–67. Epub 2013/09/24. 10.1016/j.preteyeres.2013.09.001 S1350-9462(13)00057-8 [pii]. . 24055863

[8] Charlesworth J, Kramer PL, Dyer T, Diego V, Samples JR, Craig JE, et al The path to open-angle glaucoma gene discovery: endophenotypic status of intraocular pressure, cup-to-disc ratio, and central corneal thickness. Invest Ophthalmol Vis Sci. 2010;51(7):3509–14. Epub 2010/03/20. 10.1167/iovs.09-4786 iovs.09-4786 [pii]. 20237253 PMC2904007

[9] Bian K, Murad F. What is next in nitric oxide research? From cardiovascular system to cancer biology. Nitric Oxide. 2014;43:3–7. Epub 2014/08/26. 10.1016/j.niox.2014.08.006 S1089-8603(14)00280-8 [pii]. . 25153032

[10] Stamer WD, Lei Y, Boussommier-Calleja A, Overby DR, Ethier CR. eNOS, a pressure-dependent regulator of intraocular pressure. Invest Ophthalmol Vis Sci. 2011;52(13):9438–44. Epub 2011/11/01. 10.1167/iovs.11-7839 iovs.11-7839 [pii]. 22039240 PMC3293415

[11] Lei Y, Zhang X, Song M, Wu J, Sun X. Aqueous Humor Outflow Physiology in NOS3 Knockout Mice. Invest Ophthalmol Vis Sci. 2015;56(8):4891–8. Epub 2015/08/01. 10.1167/iovs.15-16564 2422124 [pii]. . 26225628

[12] Nathanson JA, McKee M. Identification of an extensive system of nitric oxide-producing cells in the ciliary muscle and outflow pathway of the human eye. Invest Ophthalmol Vis Sci. 1995;36(9):1765–73. Epub 1995/08/01. . 7543462

[13] Chang JY, Stamer WD, Bertrand J, Read AT, Marando CM, Ethier CR, et al Role of nitric oxide in murine conventional outflow physiology. Am J Physiol Cell Physiol. 2015;309(4):C205–14. Epub 2015/06/05. 10.1152/ajpcell.00347.2014 ajpcell.00347.2014 [pii]. 26040898 PMC4537932

[14] Galassi F, Renieri G, Sodi A, Ucci F, Vannozzi L, Masini E. Nitric oxide proxies and ocular perfusion pressure in primary open angle glaucoma. Br J Ophthalmol. 2004;88(6):757–60. Epub 2004/05/19. 10.1136/bjo.2003.028357 15148207 PMC1772173

[15] Cavet ME, Vittitow JL, Impagnatiello F, Ongini E, Bastia E. Nitric oxide (NO): an emerging target for the treatment of glaucoma. Invest Ophthalmol Vis Sci. 2014;55(8):5005–15. Epub 2014/08/16. 10.1167/iovs.14-14515 55/8/5005 [pii]. . 25125670

[16] Toda N, Nakanishi-Toda M. Nitric oxide: ocular blood flow, glaucoma, and diabetic retinopathy. Prog Retin Eye Res. 2007;26(3):205–38. Epub 2007/03/06. S1350-9462(07)00005-5 [pii] 10.1016/j.preteyeres.2007.01.004 . 17337232

[17] Wink DA, Miranda KM, Espey MG, Pluta RM, Hewett SJ, Colton C, et al Mechanisms of the antioxidant effects of nitric oxide. Antioxid Redox Signal. 2001;3(2):203–13. Epub 2001/06/09. 10.1089/152308601300185179 . 11396476

[18] Ster AM, Popp RA, Petrisor FM, Stan C, Pop VI. The Role of Oxidative Stress and Vascular Insufficiency in Primary Open Angle Glaucoma. Clujul Med. 2014;87(3):143–6. Epub 2014/01/01. 10.15386/cjmed-295 cm8703p143 [pii]. 26528013 PMC4508597

[19] Lin HJ, Tsai CH, Tsai FJ, Chen WC, Tsai SW, Fan SS. Distribution of oxidation enzyme eNOS and myeloperoxidase in primary open angle glaucoma. J Clin Lab Anal. 2005;19(2):87–92. Epub 2005/03/10. 10.1002/jcla.20057 . 15756709 PMC6808135

[20] Emam WA, Zidan HE, Abdulhalim BE, Dabour SA, Ghali MA, Kamal AT. Endothelial nitric oxide synthase polymorphisms and susceptibility to high-tension primary open-angle glaucoma in an Egyptian cohort. Mol Vis. 2014;20:804–11. Epub 2014/06/19. 24940036 PMC4057245

[21] Magalhaes da Silva T, Rocha AV, Lacchini R, Marques CR, Silva ES, Tanus-Santos JE, et al Association of polymorphisms of endothelial nitric oxide synthase (eNOS) gene with the risk of primary open angle glaucoma in a Brazilian population. Gene. 2012;502(2):142–6. Epub 2012/05/09. 10.1016/j.gene.2012.04.047 S0378-1119(12)00473-8 [pii]. . 22561696

[22] Miyamoto Y, Saito Y, Nakayama M, Shimasaki Y, Yoshimura T, Yoshimura M, et al Replication protein A1 reduces transcription of the endothelial nitric oxide synthase gene containing a -786T—>C mutation associated with coronary spastic angina. Hum Mol Genet. 2000;9(18):2629–37. Epub 2000/11/07. 10.1093/hmg/9.18.2629 . 11063722

[23] Tesauro M, Thompson WC, Rogliani P, Qi L, Chaudhary PP, Moss J. Intracellular processing of endothelial nitric oxide synthase isoforms associated with differences in severity of cardiopulmonary diseases: cleavage of proteins with aspartate vs. glutamate at position 298. Proc Natl Acad Sci U S A. 2000;97(6):2832–5. Epub 2000/03/16. 97/6/2832 [pii] 10.1073/pnas.97.6.2832 10717002 PMC16015

[24] Xiang Y, Dong Y, Li X, Tang X. Association of Common Variants in eNOS Gene with Primary Open Angle Glaucoma: A Meta-Analysis. J Ophthalmol. 2016;2016:1348347 Epub 2016/06/01. 10.1155/2016/1348347 27242919 PMC4875980

[25] Kondkar AA, Azad TA, Almobarak FA, Abu-Amero KK, Al-Obeidan SA. Polymorphism rs7961953 in TMTC2 gene is not associated with primary open-angle glaucoma in a Saudi cohort. Ophthalmic Genet. 2019;40(1):74–6. Epub 2019/02/08. 10.1080/13816810.2019.1576210 . 30729851

[26] Kondkar AA, Sultan T, Almobarak FA, Kalantan H, Abu-Amero KK, Al-Obeidan SA. Plexin domain containing 2 (PLXDC2) gene polymorphism rs7081455 may not influence POAG risk in a Saudi cohort. BMC Res Notes. 2018;11(1):733 Epub 2018/10/18. 10.1186/s13104-018-3848-x [pii]. 30326957 PMC6192173

[27] Kondkar AA, Azad TA, Almobarak FA, Bahabri IM, Kalantan H, Abu-Amero KK, et al Lack of Association between Variant rs7916697 in ATOH7 and Primary Open Angle Glaucoma in a Saudi Cohort. Genet Res Int. 2018;2018:2148056 Epub 2018/12/07. 10.1155/2018/2148056 30519491 PMC6241241

[28] Kondkar AA, Azad TA, Sultan T, Al-Mobarak FA, Kalantan H, Al-Obeidan SA. Polymorphisms rs693421 and rs2499601 at locus 1q43 and their haplotypes are not associated with primary open-angle glaucoma: a case-control study. BMC Res Notes. 2019;12(1):453 Epub 2019/07/25. 10.1186/s13104-019-4491-x [pii]. 31337432 PMC6651941

[29] Ahmad SS. Controversies in the vascular theory of glaucomatous optic nerve degeneration. Taiwan J Ophthalmol. 2016;6(4):182–6. Epub 2017/10/12. 10.1016/j.tjo.2016.05.009 TJO-6-182 [pii]. 29018738 PMC5525630

[30] Yoshimura M, Yasue H, Nakayama M, Shimasaki Y, Sumida H, Sugiyama S, et al A missense Glu298Asp variant in the endothelial nitric oxide synthase gene is associated with coronary spasm in the Japanese. Hum Genet. 1998;103(1):65–9. Epub 1998/09/16. 10.1007/s004390050785 . 9737779

[31] Tunny TJ, Richardson KA, Clark CV. Association study of the 5' flanking regions of endothelial-nitric oxide synthase and endothelin-1 genes in familial primary open-angle glaucoma. Clin Exp Pharmacol Physiol. 1998;25(1):26–9. Epub 1998/03/11. 10.1111/j.1440-1681.1998.tb02138.x . 9493554

[32] Fan BJ, Liu K, Wang DY, Tham CC, Tam PO, Lam DS, et al Association of polymorphisms of tumor necrosis factor and tumor protein p53 with primary open-angle glaucoma. Invest Ophthalmol Vis Sci. 2010;51(8):4110–6. Epub 2010/04/02. 10.1167/iovs.09-4974 iovs.09-4974 [pii]. . 20357201

[33] Kang JH, Wiggs JL, Rosner BA, Hankinson SE, Abdrabou W, Fan BJ, et al Endothelial nitric oxide synthase gene variants and primary open-angle glaucoma: interactions with sex and postmenopausal hormone use. Invest Ophthalmol Vis Sci. 2010;51(2):971–9. Epub 2009/10/10. 10.1167/iovs.09-4266 iovs.09-4266 [pii]. 19815736 PMC3094851

[34] Kang JH, Wiggs JL, Rosner BA, Haines J, Abdrabou W, Pasquale LR. Endothelial nitric oxide synthase gene variants and primary open-angle glaucoma: interactions with hypertension, alcohol intake, and cigarette smoking. Arch Ophthalmol. 2011;129(6):773–80. Epub 2011/06/15. 10.1001/archophthalmol.2011.118 129/6/773 [pii]. 21670344 PMC3337676

[35] Logan JF, Chakravarthy U, Hughes AE, Patterson CC, Jackson JA, Rankin SJ. Evidence for association of endothelial nitric oxide synthase gene in subjects with glaucoma and a history of migraine. Invest Ophthalmol Vis Sci. 2005;46(9):3221–6. Epub 2005/08/27. 46/9/3221 [pii] 10.1167/iovs.05-0368 . 16123422

[36] Kosior-Jarecka E, Lukasik U, Wrobel-Dudzinska D, Kocki J, Bartosinska J, Witczak A, et al Risk Factors for Normal and High-Tension Glaucoma in Poland in Connection with Polymorphisms of the Endothelial Nitric Oxide Synthase Gene. PLoS One. 2016;11(1):e0147540 Epub 2016/01/26. 10.1371/journal.pone.0147540 PONE-D-15-33145 [pii]. 26807726 PMC4726562

[37] Jeoung JW, Kim DM, Oh S, Lee JS, Park SS, Kim JY. The Relation Between Endothelial Nitric Oxide Synthase Polymorphisms and Normal Tension Glaucoma. J Glaucoma. 2017;26(11):1030–5. Epub 2017/08/05. 10.1097/IJG.0000000000000751 . 28777225

[38] Weiss J, Frankl SA, Flammer J, Grieshaber MC, Hollo G, Teuchner B, et al No difference in genotype frequencies of polymorphisms of the nitric oxide pathway between Caucasian normal and high tension glaucoma patients. Mol Vis. 2012;18:2174–81. Epub 2012/08/25. 22919264 PMC3425578

[39] Sakai T, Shikishima K, Matsushima M, Kitahara K. Endothelial nitric oxide synthase gene polymorphisms in non-arteritic anterior ischemic optic neuropathy. Graefes Arch Clin Exp Ophthalmol. 2007;245(2):288–92. Epub 2006/04/25. 10.1007/s00417-005-0245-7 . 16633797

[40] Lay CL, Broner SW. Migraine in women. Neurol Clin. 2009;27(2):503–11. Epub 2009/03/18. 10.1016/j.ncl.2009.01.002 S0733-8619(09)00003-6 [pii]. . 19289228

[41] Liao Q, Wang DH, Sun HJ. Association of genetic polymorphisms of eNOS with glaucoma. Mol Vis. 2011;17:153–8. Epub 2011/01/20. 19 [pii]. 21245953 PMC3021569

[42] Colombo MG, Paradossi U, Andreassi MG, Botto N, Manfredi S, Masetti S, et al Endothelial nitric oxide synthase gene polymorphisms and risk of coronary artery disease. Clin Chem. 2003;49(3):389–95. Epub 2003/02/26. 10.1373/49.3.389 . 12600950

[43] Matsa LS, Rangaraju A, Vengaldas V, Latifi M, Jahromi HM, Ananthapur V, et al Haplotypes of NOS3 gene polymorphisms in dilated cardiomyopathy. PLoS One. 2013;8(7):e70523 Epub 2013/08/08. 10.1371/journal.pone.0070523 PONE-D-12-31516 [pii]. 23923002 PMC3726655

[44] Sandrim VC, Coelho EB, Nobre F, Arado GM, Lanchote VL, Tanus-Santos JE. Susceptible and protective eNOS haplotypes in hypertensive black and white subjects. Atherosclerosis. 2006;186(2):428–32. Epub 2005/09/20. S0021-9150(05)00521-6 [pii] 10.1016/j.atherosclerosis.2005.08.003 . 16168996

[45] Kondkar AA, Azad TA, Almobarak FA, Kalantan H, Sultan T, Alsabaani NA, et al Polymorphism rs10483727 in the SIX1/SIX6 Gene Locus Is a Risk Factor for Primary Open Angle Glaucoma in a Saudi Cohort. Genet Test Mol Biomarkers. 2018;22(1):74–8. Epub 2017/12/01. 10.1089/gtmb.2017.0159 . 29190129

[46] Abu-Amero KK, Kondkar AA, Mousa A, Almobarak FA, Alawad A, Altuwaijri S, et al Analysis of Cyclin-Dependent Kinase Inhibitor-2B rs1063192 Polymorphism in Saudi Patients with Primary Open-Angle Glaucoma. Genet Test Mol Biomarkers. 2016;20(10):637–41. Epub 2016/10/18. 10.1089/gtmb.2016.0140 . 27541204

[47] Abu-Amero KK, Kondkar AA, Mousa A, Osman EA, Al-Obeidan SA. Lack of association of SNP rs4236601 near CAV1 and CAV2 with POAG in a Saudi cohort. Mol Vis. 2012;18:1960–5. Epub 2012/08/10. 22876122 PMC3413415

[48] Kondkar AA, Azad TA, Almobarak FA, Kalantan H, Sultan T, Al-Obeidan SA, et al Polymorphism rs11656696 in GAS7 Is Not Associated with Primary Open Angle Glaucoma in a Saudi Cohort. Genet Test Mol Biomarkers. 2017;21(12):754–8. Epub 2017/10/13. 10.1089/gtmb.2017.0147 . 29022762

[49] Kondkar AA, Mousa A, Azad TA, Sultan T, Alawad A, Altuwaijri S, et al Polymorphism rs7555523 in transmembrane and coiled-coil domain 1 (TMCO1) is not a risk factor for primary open angle glaucoma in a Saudi cohort. J Negat Results Biomed. 2016;15(1):17 Epub 2016/10/01. 10.1186/s12952-016-0060-1 [pii]. 27687253 PMC5043619

[50] Polak K, Luksch A, Berisha F, Fuchsjaeger-Mayrl G, Dallinger S, Schmetterer L. Altered nitric oxide system in patients with open-angle glaucoma. Arch Ophthalmol. 2007;125(4):494–8. Epub 2007/04/11. 125/4/494 [pii] 10.1001/archopht.125.4.494 . 17420369

[51] Wang XL, Sim AS, Wang MX, Murrell GA, Trudinger B, Wang J. Genotype dependent and cigarette specific effects on endothelial nitric oxide synthase gene expression and enzyme activity. FEBS Lett. 2000;471(1):45–50. Epub 2000/04/13. S0014-5793(00)01356-9 [pii] 10.1016/s0014-5793(00)01356-9 . 10760510

[52] Naber Ch K, Frey UH, Oldenburg O, Brauck K, Eggebrecht H, Schmermund A, et al Relevance of the NOS3 T-786C and G894T variants for cholinergic and adrenergic coronary vasomotor responses in man. Basic Res Cardiol. 2005;100(5):453–60. Epub 2005/07/21. 10.1007/s00395-005-0530-y . 16032374

[53] Fischmann TO, Hruza A, Niu XD, Fossetta JD, Lunn CA, Dolphin E, et al Structural characterization of nitric oxide synthase isoforms reveals striking active-site conservation. Nat Struct Biol. 1999;6(3):233–42. Epub 1999/03/13. 10.1038/6675 . 10074942

[54] Joshi MS, Bauer JA. Preliminary computational modeling of nitric oxide synthase 3 interactions with caveolin-1: influence of exon 7 Glu298Asp polymorphism. Acta Biochim Biophys Sin (Shanghai). 2008;40(1):47–54. Epub 2008/01/09. 10.1111/j.1745-7270.2008.00369.x . 18180853

[55] Godfrey V, Chan SL, Cassidy A, Butler R, Choy A, Fardon T, et al The functional consequence of the Glu298Asp polymorphism of the endothelial nitric oxide synthase gene in young healthy volunteers. Cardiovasc Drug Rev. 2007;25(3):280–8. Epub 2007/10/09. CDR017 [pii] 10.1111/j.1527-3466.2007.00017.x . 17919260

[56] Abu-Amero KK, Kondkar AA, Mousa A, Osman EA, Al-Obeidan SA. Decreased total antioxidants in patients with primary open angle glaucoma. Curr Eye Res. 2013;38(9):959–64. Epub 2013/05/09. 10.3109/02713683.2013.794246 . 23651069

[57] Fairchild TA, Fulton D, Fontana JT, Gratton JP, McCabe TJ, Sessa WC. Acidic hydrolysis as a mechanism for the cleavage of the Glu(298)—>Asp variant of human endothelial nitric-oxide synthase. J Biol Chem. 2001;276(28):26674–9. Epub 2001/05/02. 10.1074/jbc.M103647200 M103647200 [pii]. . 11331296

[58] Hingorani AD. Polymorphisms in endothelial nitric oxide synthase and atherogenesis: John French Lecture 2000. Atherosclerosis. 2001;154(3):521–7. Epub 2001/03/21. S0021-9150(00)00699-7 [pii]. 10.1016/s0021-9150(00)00699-7 . 11257252

[59] Golser R, Gorren AC, Mayer B, Schmidt K. Functional characterization of Glu298Asp mutant human endothelial nitric oxide synthase purified from a yeast expression system. Nitric Oxide. 2003;8(1):7–14. Epub 2003/02/15. S1089860302001313 [pii]. 10.1016/s1089-8603(02)00131-3 . 12586536

[60] Wang J, Dudley D, Wang XL. Haplotype-specific effects on endothelial NO synthase promoter efficiency: modifiable by cigarette smoking. Arterioscler Thromb Vasc Biol. 2002;22(5):e1–4. Epub 2002/05/15. 10.1161/01.ATV.0000016248.51577.1F 12006409 PMC1350055

